# The effect of solid food diet therapies on the induction and maintenance of remission in Crohn’s disease: a systematic review

**DOI:** 10.1186/s12876-024-03315-7

**Published:** 2024-08-06

**Authors:** Jennifer Li Zhang, Nikil Vootukuru, Olga Niewiadomski

**Affiliations:** 1 Department of Gastroenterology, Eastern Health, Melbourne, VIC Australia; 2 Monash University, Melbourne, VIC Australia

**Keywords:** Induction, Remission, Crohn’s disease, Solid food, Diet

## Abstract

**Background:**

The efficacy of highly restrictive dietary therapies such as exclusive enteral nutrition (EEN) in the induction of remission in Crohn’s disease (CD) are well established, however, ongoing issues exist with its poor palatability, restrictions, and adherence. The primary aim of this review is to evaluate the current evidence for the efficacy of exclusively solid food diets on the induction and maintenance of clinical and biochemical remission in CD. Secondary aims include impact on endoscopic healing and quality of life.

**Methods:**

A systematic review of all randomised controlled trials (RCTs), open-label randomised trials and head-to-head clinical trials assessing solid food diet intervention in patients with active or inactive Crohn’s disease was conducted. Studies included adult and paediatric patients with a verified disease activity index at baseline and follow up (Harvey Bradshaw Index, HBI; Crohn’s disease activity index, CDAI and paediatric CDAI, PCDAI). Additional secondary endpoints varied between studies, including endoscopic and biochemical responses, as well as quality of life measures. Two authors independently performed critical appraisals of the studies, including study selection and risk of bias assessments.

**Results:**

14 studies were included for review, with several studies suggesting clinically significant findings. Clinical remission was achieved in a paediatric population undertaking the Mediterranean diet (MD) (moderate risk of bias). In adults, the Crohn’s disease exclusion diet (CDED) was comparable to the CDED with partial enteral nutrition (PEN) diet in induction of remission (moderate risk of bias). A low fermentable oligosaccharides, disaccharides, monosaccharides and polyols (FODMAP) diet was also shown to decrease symptoms in patients with quiescent or mildly active CD (high risk of bias), however, this was not corroborated by other low FODMAP diet studies.

**Conclusions:**

There are promising outcomes for the MD and CDED in inducing clinical remission in mild to moderate CD. The results need to be interpreted with caution due to design limitations, including issues with combining outcomes among CD and UC patients, and small sample size. The current evidence for solid food dietary therapy in CD is limited by the lack of high quality studies and moderate to high bias. Future well designed studies are needed to confirm their efficacy.

**Supplementary Information:**

The online version contains supplementary material available at 10.1186/s12876-024-03315-7.

## Background

The presumed pathogenesis of Crohn’s disease (CD) is the interplay between environmental factors and the gut microbiome in genetically predisposed individuals [1–4] that results in a dysregulated immune system and inflammation. Dietary factors are considered one of the most significant of these environmental factors, as these are key in shaping the composition and function of the gut microbiota [5, 6]. The rising prevalence and incidence of CD in Western countries, and more recently in previously low prevalence countries adopting a Westernised lifestyle, have coincided with significant shifts in diet [7, 8]. These shifts have included a diet high in refined carbohydrates, sugars, and processed meat. This raises the possibility of diet as a causative factor in CD.

Current management of CD focuses on inducing short-term remission and maintaining long-term remission with medical therapy. Disease activity is closely monitored to ensure patients remain in remission, as it is now recognised that chronic activity leads to poor outcomes and complications. Monitoring methods include clinical evaluation based on symptoms and validated clinical indices, as well as objective inflammatory markers such as C-reactive protein (CRP) and faecal calprotectin (FCP). Imaging studies and endoscopy are used to confirm efficacy of therapies and disease remission.

Exclusive enteral nutrition (EEN) has been established as an alternative therapy to medications for inducing remission in CD. The efficacy of the EEN diet is well established for the treatment of established CD, specifically the induction of remission in those with active disease. It involves substituting all food with liquid formulas. Guidelines recommend the use of EEN as a first-line treatment in paediatric patients [9–11]. However, challenges exist due to its poor palatability, restrictive nature, low long-term tolerance, and compliance [12].

In addition, the role of diet as maintenance therapy for patients with inactive disease, either as an adjunct to medication or as monotherapy to prevent disease relapse, remains unknown. Stringent diets such as EEN are not a feasible option long term due to the limitations outlined around tolerability. Partial enteral nutrition (PEN), which involves 50% caloric intake from liquid formula and 50% from a restricted diet, has shown some promise in both inducing and maintaining remission in systemic meta-analyses [13, 14], although variability in outcomes remains a concern. PEN remains restrictive and long-term tolerability is a problem. Less restrictive dietary therapies are needed, especially for those with inactive disease, if this is to become a viable maintenance therapy option [15].

Previous systematic reviews evaluating dietary interventions in CD have included both solid food diets with a liquid formula-based component, such as PEN and EEN. Since then, there is growing evidence for solid food diet therapies in managing symptoms in CD patients and as an adjunct to medical therapy [16–19], which can readily be integrated into clinical practice for patients seeking dietary guidance from their physician. It is important that this is grounded in high-quality evidence, particularly given the prevalent misinformation that patients encounter.

In this review, we focus on solid food dietary therapy interventions, as a guide for gastroenterologists to draw on within their clinical practice. Our aims were to systematically review prospective randomised clinical trials that compared solid food diets with a control diet or another dietary intervention, in the induction and/or maintenance of remission in CD; and to grade the quality of evidence.

## Methods

This review was based on the Joanna Briggs Institute’s (JBI) framework for systematic reviews and written in accordance with the Preferred Reporting Items for Systematic Reviews and Meta-Analyses (PRISMA).

### Data sources and search strategy

A comprehensive database search of MEDLINE, EMBASE, Cochrane, ICTRP (WHO), ANZCTR, ClinicalTrials.gov, MEDNAR and BMC was conducted on 11 November 2023. Keywords and search strings relevant to the topic were searched under the fields “Article Title” and “Abstract”, and where possible, medical subject headings (MeSH) were used. The following MeSH terms were included in the MEDLINE search: Crohn’s disease, diet, remission, and induction (see Appendix 1 for full search). The search strategy employed for MEDLINE was adapted for the other databases. References of key articles were examined to identify further relevant publications. There were no limitations placed on the time frame of included studies.

### Study selection

Randomised controlled trials (RCTs) and prospective controlled trials involving solid oral diets were included. Head-to-head trials with no control were also included. Studies including PEN and EEN were only included if the comparator consisted of solid food diets. Adult and paediatric patients were included, regardless of age, location, or disease remission status.

Studies included baseline and follow up validated clinical disease indices, including Harvey Bradshaw Index (HBI), Crohn’s Disease Activity Index (CDAI), Inflammatory Bowel Disease Questionnaire (IBDQ) and Simple Endoscopic Score for Crohn’s Disease (SES-CD). There were no parameters set on publication date or language. Conference abstracts, opinion letters and editorials were excluded due to limited information. Articles were excluded if oral diet modifications involved nutrient supplementation, probiotics, liquid diets, or medical foods.

### Title and abstract review

Two reviewers (JZ, NV) independently screened titles and abstracts for inclusion and retrieved relevant full-text articles. Any disagreements between the two reviewers were resolved by discussion with a third reviewer (ON). Multiple reports of the same study were collated and reported as a single study, as appropriate.

### Data extraction

The following data were extracted from the included studies following the full-text review and documented into an Excel spreadsheet.

- Year of publication, country of study, study design.
- Participants: number and age of patients.
- Description of the control and intervention.
- Outcome measures, time points and results.

Extracted data was cross-checked by authors JZ and NV.

### Critical appraisal

Included randomised and non-randomised controlled trials were critically appraised for risk of bias using the Cochrane Risk of Bias tool (RoB 2) [20]. Two reviewers (JZ, NV) independently conducted this appraisal and resolved any disagreements through discussion. Given the variability of study designs, total scores of included papers are intended as a relative judgement of methodological quality.

## Results

### Characteristics of eligible studies

**Fig. 1:**
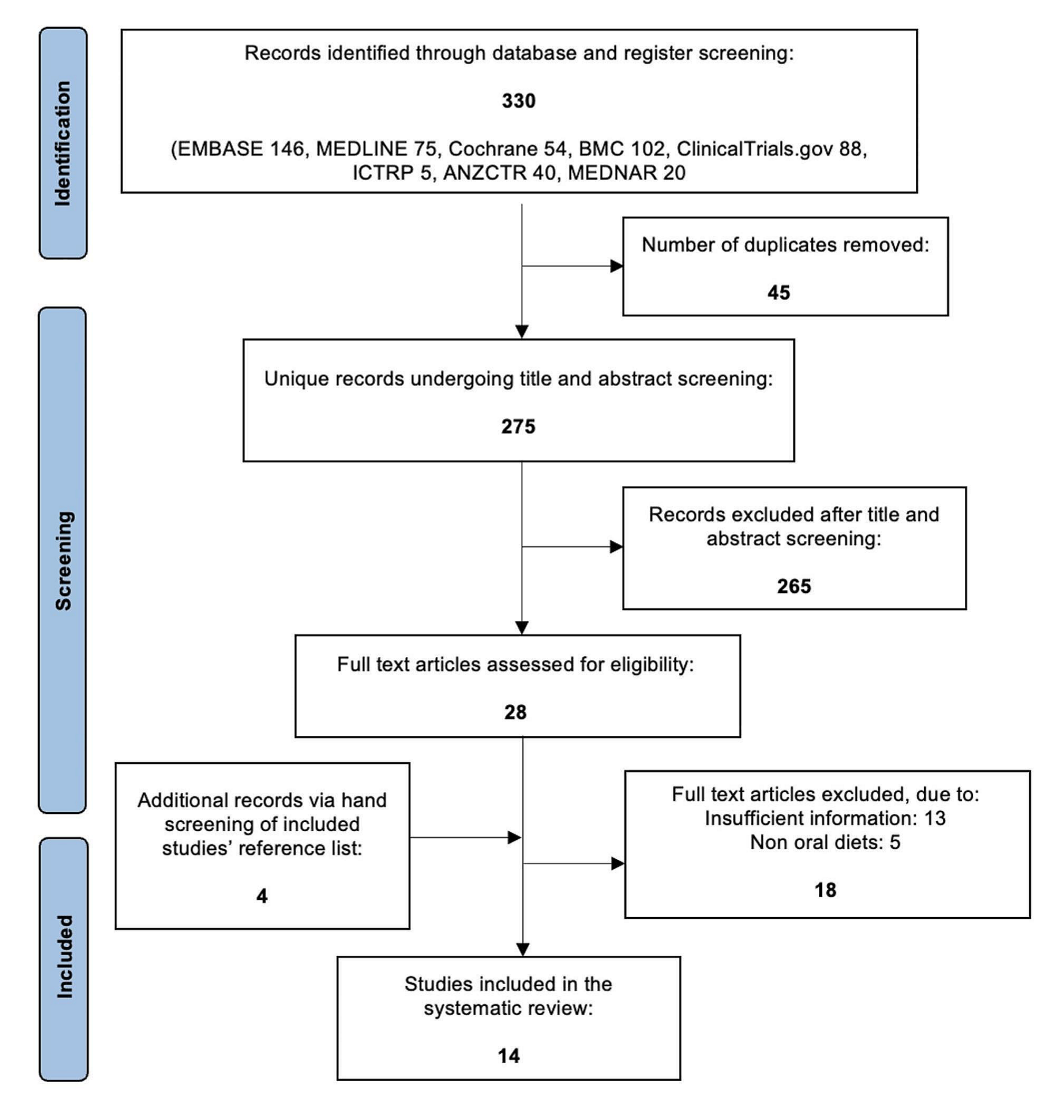
PRISMA flow diagram

The full search identified 530 records, of which 28 were selected for full-text review after title and abstract screening (Fig. 1: PRISMA flow diagram). Of these, 13 were excluded due to insufficient information (abstracts or letters to editors), and five were excluded due to not strictly incorporating a solid food diet. Four further articles were identified on reference review of key articles. Ultimately, there were 14 studies [11 RCTs, two head-to-head randomised trials, and one open label randomised trial] that met the inclusion criteria **(**Tables 1 and 2**).** Patients who completed the duration of intervention and adhered to treatment were included in the outcome. Patients who experienced a clinical relapse prior to the end of study duration were also included. Single arm studies were excluded given the lack of adequacy in distinguishing outcomes from the natural evolution of disease activity.

**Table 1:** Outcomes for patients with quiescent or mildly active CD

Author Country (Year)	Study Design	Participant Age	Study Duration	Study Sample Size	Disease Status at Initiation of Study	Intervention (ITT unless specified)	Control (ITT unless specified)	Outcome Measures	Disease activity or relapse after remission (Results)	Inflammatory markers (Results)	Quality of life (Results)	Study authors’ conclusions
***Cox et al*** ***London*** ***(2020)***	RCT	≥ 18 years	4 weeks	ITT analysis (n = 26) PP analysis (n = 43 including CD + UC patients combined) Withdrawal after study commencement (CD + UC combined): 6 participants (2 due to non-consent, 1 became pregnant, 2 commenced steroids due to an IBD flare, 1 commenced antibiotics for an unrelated infection). Of the 46 patients (CD + UC total) completing the trial, 3 were non-compliant with their diet, leaving 43 participants (21 low FODMAP diet, 22 sham diet).	Patients with quiescent CD, experiencing ongoing gut symptoms (IBS-M, IBS-D, or IBS-U) and naïve to low FODMAP diet. Patients with dose changes of CD specific medications in the preceding 4 to 12 weeks* or antibiotics in the preceding 8 weeks were excluded. *See article for time frames for specific medications	Low FODMAP diet (n = 14)	Sham diet (n = 12)	**1. Gut symptoms and HR-QOL** Measured using IBS-SSS, stool frequency and consistency. **2. Disease activity** Measured using HBI. **3. Changes in inflammatory markers** Measured using CRP and FCP	No difference in HBI score between low FODMAP and sham diet at end of trial (p = 0.814)	No difference in end of trial FCP and CRP.	No difference in change in IBS-SSS score (including pain severity, days of pain, bloating severity, satisfaction with bowels, impact on life) following low FODMAP compared with sham diet (p = 0.515) or in end-of-trial IBS-SSS score (p = 0.515). The stool frequency was significantly improved in patients with low FODMAP diet (p = 0.019), but not consistency, as measured by the Bristol stool scale.	In patients with quiescent CD, there was no significant difference after 4 weeks in change in irritable bowel syndrome severity scores, nor other markers of disease activity and inflammation.
***Bodini et al*** ***Italy*** ***(2019)***	RCT	18–80 years	6 weeks T0 – initial T1 – end of 6 week intervention	ITT analysis (n = 35) PP analysis (same as ITT) Withdrawal after study commencement: Nil	Patients in remission or mild disease activity, as assessed by HBI < 8 in patients with CD. Including patients with functional GI symptoms that meet Roma IV criteria for the diagnosis of IBS; and stable CD therapy with no modification of treatment within at least the 12 week period before enrolling.	Low FODMAP diet (n = 18)	Standard diet with usual FODMAP intake (n = 17)	**1. Disease activity** Measured as HBI, FCP, CRP **2. Quality of life** Measured using IBD-Q	Median HBI decreased (IQR 2–3; p = 0.024) in the LFD group but not in the SD group (IQR 2–4; p = 0.322). In the CD + UC cohorts combined, there was a statistically significant decrease in median FCP values in the LFD group but not in the SD group at T0 and T1 (p = 0.004). There was no statistically significant difference between median CRP values at T0 and T1 (p = 0.719).	Not applicable	In the CD + UC cohorts combined, there was a statistically significant increase in median IBD-Q in the LFD group (p = 0.05). The difference between the groups at T1 was not significant (p = 0.886).	Limitation in results due to the study combining data for both CD and UC in terms of outcome significance.
***Albenberg et al*** ***USA North Carolina*** ***(2019)***	RCT	> 18 years	49 weeks	Modified ITT analysis (n = 202) PP analysis (same as modified ITT) Withdrawal after study commencement: Nil, however the 11 patients who did not complete follow-up surveys were not included in the final analysis (i.e. modified ITT)	Patients with CD in symptomatic remission, defined by sCDAI ≤ 150. Patients with a history of steroid use within prior 2 weeks were excluded.	Less than one serving/month of red or processed meat (n = 87)	Two servings/week of red or processed meat (n = 115)	**1. Relapse after remission** Defined as increase in sCDAI score by ≥ 70 points and to > 150, or a need for CD surgery, or new CD medication. Moderate or severe relapse was based on an increase in sCDAI of > 219. **2. Faecal calprotectin** DHQ II questionnaire at week 1 to assess baseline dietary pattern. Outcome of study recorded at week 49, or at time of relapse (if earlier).	Any and moderate to severe relapse occurred in 62% of participants in the high-meat group and 42% of participants in the low-meat group. There were no significant differences in time to any (p = 0.61) or moderate/severe (p = 0.50) relapse.	At week 20, 18 participants in each arm submitted a stool sample for faecal calprotectin. There was no statistically significant difference (p = 0.13) between the higher meat arm compared the low meat arm.	Not applicable	Among patients with CD in remission, level of red and processed meat consumption was not associated with time to symptomatic relapse.
***Pederson et al*** ***Denmark*** ***(2017)***	RCT	20–70 years	6 weeks	ITT analysis (n = 28) PP analysis (n = 78 including CD + UC patients combined) Withdrawal after study commencement (CD + UC combined): 7 patients (LFD) due to difficulty with diet and 4 patients (habitual diet) due to lack of compliance with surveys.	Patients in remission or with mild-moderate disease activity, and co-existing IBS like symptoms with baseline IBS-SSS of at least 75 points. Patients required to be on maintenance therapy with 5-ASA, AZA or biologicals or a combination. Patients with a history of steroid use within prior 4 weeks were excluded.	Low FODMAP diet (n = 14)	Standard Diet (n = 14)	**1. Quality of life** Measured using IBS-SSS, SIDBQ and IBS-QOL questionnaire **2. Disease activity** Measured using HBI, CRP, FCP	No significant reduction of HBI observed for those on LFD vs habitual diet (p = 0.09). Nil significant correlation between HBI and IBDQ (p = 0.09).	Not applicable	Significantly greater IBS-SSS reduction with LFD than those on habitual diet (p = 0.02) In CD and UC cohorts combined, a statistically significant improvement in SIDBQ was observed in those on LFD when compared to those on a habitual diet (p < 0.01). IBS-QOL did not improve significantly in either the LFD or habitual diet group (p = 0.09).	A low FODMAP diet can reduce IBS-like symptoms and possibly increase quality of life in patients with CD in remission. There were limitations due to the combining of CD and UC data when assessing for FC remission.
***Lorenz-Meyer et al*** ***Germany*** ***(1996)***	RCT	18–70 years	12 months	ITT analysis (n = 134) PP analysis (n = 102) Withdrawals after study commencement: 9 patients in the diet group and 2 patients in the control group due to relapse. The remaining 21 patients due to non-compliance (unknown reason).	Patients with active CD (CDAI > 200) were recruited and included once they reached remission (CDAI ≤ 150) under conventional steroid therapy over a 3 month period. Patients who were taking medications for treatment of CD were excluded. All patients were given low-dose prednisolone during the first 8 weeks.	Low carbohydrate diet of < 84g/day (n = 69 for ITT) (n = 44 for PP) *Not included in this review: Active treatment group (n = 70)* *5g/day of highly concentrated omega-3 fatty acid compound, taken via capsules.*	Placebo capsules in addition to habitual diet (n = 65 for ITT) (n = 58 for PP)	**1. Relapse after remission** CDAI and CRP were used as criteria for relapse. A relapse was defined as an increase of CDAI above 200 points by at least 60 points above baseline, plus an increase of the CRP by two standard deviations above the mean of the healthy population in the respective centres. CDAI was calculated at regular time intervals (1, 2, 3, 6, 9 and 12 months).	No difference between low carbohydrate and placebo groups (p = 0.38) on an ITT analysis. Patients did gain benefit (53%; p = 0.023) for as long as they maintained the low carbohydrate diet (approximately 225 days out of the 365 day duration of study).	Not applicable	Not applicable	A low carbohydrate diet did not decrease relapses after remission was sustained. Given the difference between PP and ITT analyses, the time point at which patients admitted to not keeping to their diet may have been close to the time of relapse. The question remains whether the non-compliant patients dropped out early because they sensed a relapse approaching or whether their condition deteriorated because they failed to comply with the diet.
***Ritchie et al*** ***London *** ***(1987)***	RCT	Nil age range specified	2 years	ITT analysis (n = 352) PP analysis (N/A) Withdrawals after study commencement: 178 patients required treatment or withdrew due to non-compliance and other unknown reasons.	Patients with inactive or mildly active CD but not taking drug treatments apart from maintenance sulfasalazine.	Natural unrefined carbohydrates only, avoiding all products containing sugar or white flour (n = 190)	Refined carbohydrate diet with unrestricted sugar intake (n = 162)	**1. Relapse after remission** Defined as the need for medical or surgical treatment in hospital; need for corticosteroid (if not already being taken), antibiotic or immunosuppressive drug.	No significant change in clinical score, stool count and body weight with either diet. Marginal significance (0.05 < p < 0.1) between both groups regarding hemicolectomy, resection of anastomosis, stricturoplasty and ileostomy.	Not applicable	Not applicable	Nil convincing difference in the clinical course of disease between the two treatment groups

### Dietary therapy in quiescent and mildly active Crohn’s disease

#### Maintenance of remission

There were six RCTs that evaluated the effect of dietary interventions in 777 adult patients in remission (or mildly active CD) as a maintenance therapy. The duration of intervention was highly variable between the studies, ranging from four weeks [21] to two years [22].

Three studies assessed the efficacy of a low FODMAP diet in quiescent or mildly active CD. Two of the studies compared a low FODMAP diet against a standard ‘control’ diet [17, 21], and one compared to the patient’s usual diet [23]. All included patients had co-existing IBS symptoms. One study included a homogenous population group of patients with inactive disease [17], the other two studies combined patients with inactive and mildly active disease at baseline [21, 23]. Disease activity was measured using the HBI. There was no difference in HBI score between the low FODMAP (3.2, SEM 0.4, *n* = 14) and control diet group within this study (3.4, SEM 0.5, *n* = 12) at four weeks (*p* = 0.814) [21]. A six-week study included patients with co-existing IBS like symptoms measured by the irritable bowel severity scoring system (IBS-SSS). No significant reduction in HBI was observed for those on a low FODMAP diet (median 3, IQR 1–5, *n* = 18) compared with those on the standard diet (median 6, IQR 3–9, *n* = 17; *p* = 0.09). These findings are in contrast with a six-week RCT, in which the median HBI decreased significantly in the low FODMAP diet group (IQR 2–3, *p* = 0.024, *n* = 18) but not in the standard diet group (IQR 2–4; *p* = 0.322, *n* = 17) [17]. The use of concomitant medication and the duration of stable dosage prior to study enrolment varied among the studies. One study enforced a stable dose of maintenance therapy with 5-aminosalicylic acid, azathioprine, or biologics [23], while in the other two studies [17, 21] this was not an inclusion criteria. Duration of dietary therapies in all three studies was short (four to six weeks) especially when looking at patients with quiescent disease and risk of relapse.

Dietary therapy as a maintenance therapy was explored in multiple studies comparing other diets, with no significant benefit seen in preventing relapse of CD [22, 24, 25]. A large RCT (*n* = 202) assessed the impact of a low meat diet on the risk of disease relapse and activity. Patients were in symptomatic remission (short Crohn’s disease activity index (sCDAI) < 150) and disease flare was defined as a sCDAI score increase by ≥ 70 and to > 150, or need for CD surgery, or new CD medication [24]. Participants were assigned to either two servings per week (*n* = 115) or less than one serving per month (*n* = 87) of red meat for the duration of 49 weeks. There was no significant difference in time to any (*p* = 0.61) or moderate-severe (*p* = 0.50) relapse.

A large study (*n* = 204) compared a low carbohydrate diet (LCD) with a control diet based on general dietary advice encouraging high fibre intake, over the course of 12 months. A third arm was included of omega three capsules but not reported in this review as this is not a solid food dietary intervention. The definition for relapse was a CDAI score increase by ≥ 60 and/or to > 200, as well as an increase of the C-reactive protein (CRP) by two standard deviations above the mean of the healthy population [25]. Patients in both arms received an eight-week course of low dose prednisolone at onset of the study. There was no difference in risk of relapse between the two diet strategies on an intention to treat (ITT), with nine and two patients from the intervention and control arm withdrawing prematurely due to relapse, respectively. Additionally, only 15.9% were able to adhere to the LCD in its full at 12 months, and of the patients that did adhere, 53% of patients did not relapse.

In another RCT (*n* = 352) with a long study duration of two years [22], a refined carbohydrate diet consisting of white flour, rice and unrestricted sugar intake was compared with a natural unrefined carbohydrate diet. The latter avoided all products containing sugar or white flour and included wholegrains and legumes. The unrefined carbohydrate diet was based on a previously published prospective Bristol cohort study [26], in which it appeared to improve the prognosis of patients with CD, decreasing the need for hospital treatment and surgery. In the current study, there was no difference in risk of relapse as assessed by clinical scores, stool frequency and need for surgery There were twenty patients (10.5%) who withdrew from the unrefined carbohydrate arm due to non-compliance, compared to four (2.5%) in the refined carbohydrate arm. Dropout rates were high, at over 50% by two years (178 patients of 352) either due to relapse, non-compliance, or other unknown reasons. It was unclear if earlier drop out was due to onset of symptoms or whether their condition deteriorated because of non-compliance to the diet. At study conclusion, 66 (34.7%) patients were in remission as compared with 52 (32.1%), in the refined and unrefined carbohydrate diet, respectively with no statistical difference. There was no change in inflammatory markers.

#### Impact of dietary therapy on quality of life

Quality of life was measured in one of the FODMAPs studies with the short inflammatory bowel disease questionnaire (SIDBQ) score. Results were a combined analysis of CD and ulcerative colitis (UC) patients. Quality of life improved in the IBD group [17] with no sub-group analysis between UC and CD.

### Dietary therapy in active Crohn’s disease

#### Induction of remission

There were seven RCTs which assessed the impact of solid food dietary therapy on induction of remission in active CD, two of which exclusively included children and/or adolescents [16, 27].

El Amrousy et al. conducted a 12-week study in 54 paediatric CD patients with mild to moderate disease activity (paediatric Crohn’s disease activity index (PCDAI) 10–45). All patients required a stable immunomodulator and biologic dose for four and eight weeks prior to study entry, respectively. The MD group (*n* = 26) demonstrated clinical remission in 14 patients compared to only eight patients in the habitual diet (*n* = 28; *p* = 0.04) after 8 weeks of therapy. By week 12, clinical remission rates were higher in the MD group, supported by a lower mean PCDAI score (*p* = 0.02) [16]. Biochemical and inflammatory markers including CRP and FCP were combined with UC rather than individually for the two IBD subtypes of CD and UC.

A group of 14 paediatric patients with mild to moderate CD were randomised to one of three diets for the study duration of 12 weeks. These groups included the simple carbohydrate diet (SCD; excludes grains, milk, sugars and processed foods), the modified SCD diet (includes oats and rice) and a whole food diet (eliminates wheat, corn, sugar, milk and food additives) [27]. There were five, five and four patients respectively in each group. The ten patients who completed the study demonstrated clinical remission at week 12, with no obvious difference in the intention to treat (ITT) and per-protocol (PP) between the dietary arms of the study. However, tests of statistical significance were not undertaken due to small sample sizes and there was no control arm.

A study comparing a low IgG4 diet to a sham diet (*n* = 98) for a total of four weeks showed improved clinical remission rates. The intervention low IgG4 diet excluded foods based on the measurement of IgG4 titres to various food exposures, showing best improvement when excluding foods with the four highest IgG titres, namely milk, beef, pork, and eggs. The sham control diet excluded the four foods that correlated with the lowest IgG4 levels. No medication changes were allowed in the eight weeks leading up to the study. After four weeks of treatment, there was a statistically significant reduction in CDAI by a mean of 41 points in the treatment arm (*p* = 0.009) [28], as compared to the sham arm. There was no significant difference in biochemical markers of inflammation, including CRP and FCP.

A 1985 study comparing low residue (fibre) diet to a standard diet over two years showed no significant rates of clinical remission, measured using CDAI, at the end of the study period [29]. Other disease outcomes included requirement for surgery or hospitalisation and new complications, however there was insufficient data within the inactive group to draw any conclusions for these secondary outcomes. Compliance rates were not reported.

A small study of 14 patients with mild to moderately active CD (CDAI 150–220) randomised patients to two dietary interventions. The therapeutic arm had a complex dietary intervention for six weeks with emphasis on farm sourced organic food (including red meat consumption with specific oil and breads), comparing this to a low fat and high carbohydrate diet. After six weeks, disease activity was reduced in both groups with no significant difference. Endoscopic healing was achieved in 75% (three of four patients) of the active arm, compared to one of nine in the control (*p* = 0.027) [30].

The Crohn’s disease exclusion diet (CDED) (*n* = 21) with PEN was compared to CDED alone (*n* = 19) in adults with mild to moderately active CD. The CDED is a complex three phase diet that mandates five foods to be consumed daily to provide specific fibres, starches and protein while restricting animal and dairy food items along with wheat and processed foods. Participant selection was stringent, resulting in a homogenous population. Inclusion criteria included clinical activity scores with an objective measure of inflammation (colonoscopy, imaging, or inflammatory marker elevation). This study showed comparable six-week clinical remission rates of 68% (13 of 19 patients) in the CDED with PEN arm and 57% (12 of 21 patients) in the CDED only arm (*p* = 0.462). [18]. Of those who responded at week six, 80% were in sustained remission by week 24, with no difference between the two treatment arms. Baseline markers of inflammation were measured as secondary outcomes in all patients (585 ug/L for CDED with PEN, and 325 ug/L for CDED). By week 12, the calprotectin had reduced in both arms (median 104.1 for CDED with PEN and 97.3 for CDED, *p* = 0.599). A similar pattern was seen with CRP. There was no control diet in this study, but the CDED with PEN has previously been compared to EEN (gold standard dietary therapy in CD), with equal efficacy [31]. This was a small pilot study with favourable outcomes, but limited by sample size and was therefore underpowered.

A large head-to-head randomised study (*n* = 191) [32] compared the Specific Carbohydrate Diet (SCD) with the Mediterranean Diet (MD), in the DINE CD study in a refractory group of patients (> 60% had previously trialled biologics) with long duration of disease (median of 10 years). The SCD eliminates all grains, sugars, processed foods and restricts dairy to hard cheese and fermented yoghurt. On the other hand, the MD incorporates whole grain along with plant based and fibre foods, limiting red meat. The study was designed as a superiority study, hypothesizing that the SCD diet was superior. The primary end point was not met, as there was no significant difference in clinical remission rates between the two diets at week 6 (SCD 46.5%, MD 43.5%, *p* = 0.77) as defined by a CDAI < 150). There was an improvement in disease activity as measured by the sCDAI, CDAI, and patient reported outcomes inclusive of quality of life, measured by the short inflammatory bowel disease questionnaire (sIBDQ), fatigue, sleep interference, pain, and social isolation (*p* < 0.02) in both arms. Biochemical markers were only available in a minority of patients. Those with an elevated calprotectin at baseline (36 patients), 33% had a reduction (to < 250 ug/L and a decrease of > 50% from baseline), but there was no difference in inflammatory markers between the MD and SCD arms. A lack of placebo or control group is a limitation in this study. Adherence was self-reported only, with rates of 68% and 64% at week 6, and 40% and 42% at week 12 in the SCD and MD arms.

### Quality of life

Quality of life was measured using IBDQ in four studies, in which it was significantly improved in the intervention group in three studies [28, 32, 33], one being a diet excluding foods with the highest IgG titres; the other being a diet high in fibre and low refined carbohydrate diets and the third, in both dietary treatment arms inclusive of the SCD and MD. Degree of improvement in this third group was equally significant.

### Endoscopic remission

Endoscopic remission was assessed in two studies with favourable outcomes. In the CDED and CDED + PEN study, endoscopic assessment using the SES-CD score was available in 29 of 44 patients at baseline [18]. Of these, 22 patients had paired colonoscopies from baseline to week 24, and showed the median SES-CD reduced by a median of five points from baseline in all patients (*p* = 0.0025). There was no significant difference in the proportion of patients who achieved endoscopic remission between the two groups (*p* = 0.7047). The second study assessing endoscopic response was strictly an organic food study with red meat [30], finding an improvement of intestinal lesions (*p* = 0.027) compared to the control group.

**Fig. 2:**
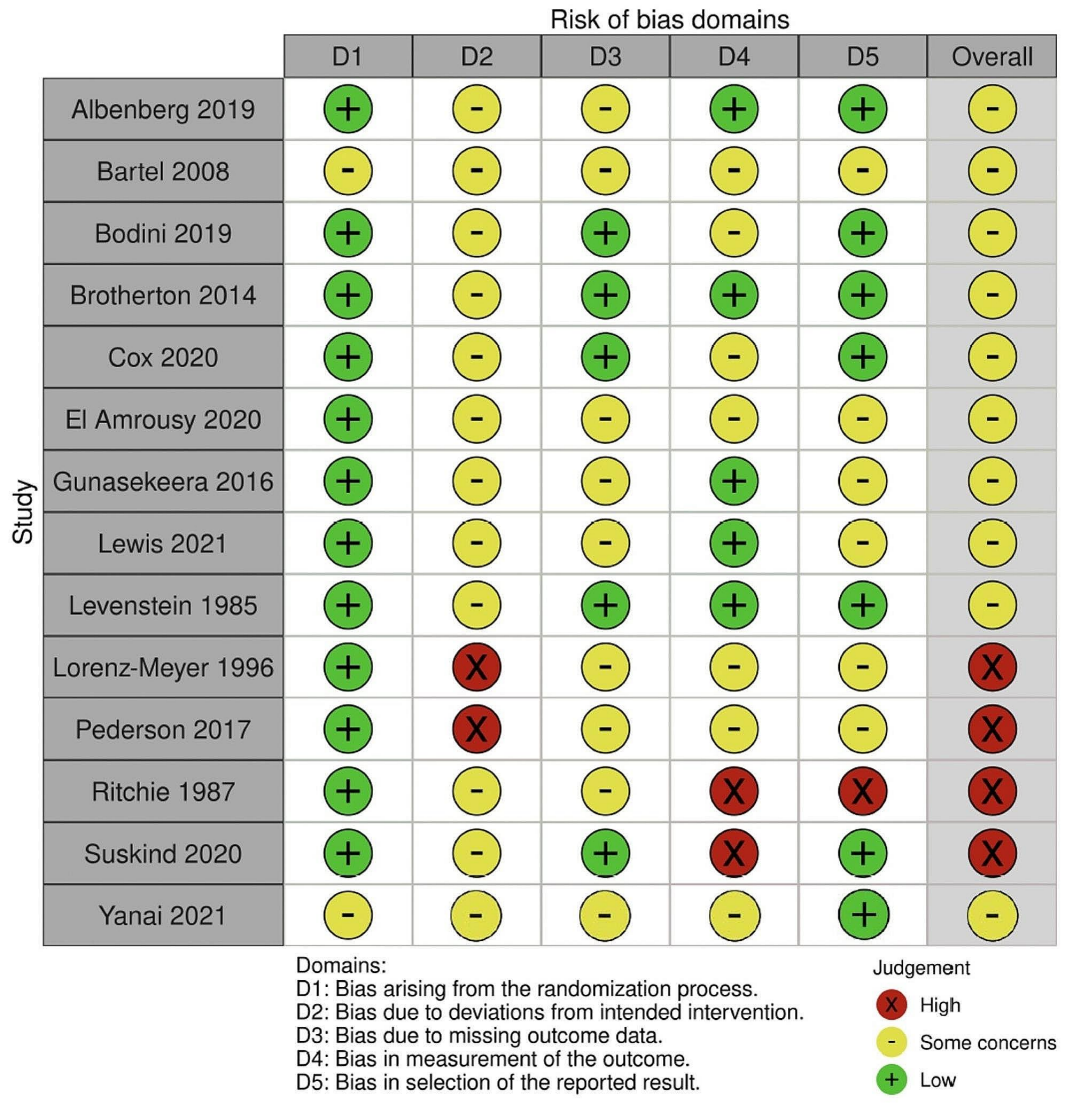
Risk of bias

This review utilised the Cochrane RoB 2 tool [20] to evaluate the bias in judgement for all 14 included studies. Either a moderate or high degree of concern was noted overall, with bias across all domains, most notably in deviations from intended intervention and bias in measurement of outcome (Fig. 2).

## Discussion

This is the first systematic review to compare the clinical, biochemical, and endoscopic efficacy in solid food dietary therapies in inducing and maintaining clinical remission in CD, as well as the impact of solid food diets on quality of life. Previous systematic reviews that assessed dietary therapies in CD incorporated liquid diets and food substitutes, which are limited in their adaptability to long term therapy due to poor palatability, low adherence and tolerance [6]. This review focuses exclusively on solid food diets to help the healthcare professional navigate one of the most frequently posed questions by patients with CD: “How can diet impact CD?” and “What should I eat?” It aims to provide the backbone for practical evidence-based dietary advice that can be offered in the consulting room to CD patients.

### Quiescent or mildly active CD (maintenance therapy)

From the six studies of over 700 patients that assessed efficacy of solid food diets in adult patients with mild or quiescent CD [17, 21–25], only one low FODMAP diet showed better symptom control and an improvement in quality of life, although a combined outcome of CD and UC was reported [23].

The low FODMAP diet is an attractive dietary therapy in CD due to its established success in irritable bowel syndrome [34], a common gastrointestinal condition which can have a similar symptom profile to CD. The role of the low FODMAP diet in CD has not been clearly established previously [35]. The positive findings from one of three studies reviewed are favourable but this was not confirmed with an improvement in the inflammatory markers. Given IBS is not uncommon in CD, it remains unclear if symptomatic benefit was due to benefit to underlying co-existing IBS or CD activity. There are limitations in the heterogenous inclusion criteria within these studies, such as differing disease activities at baseline and concomitant medication use, in addition to the results being displayed as a combined end point for both CD and UC patients [21]. Sample size was small across all the studies (26 to 35 patients). Follow up time was also short in all studies (up to 3 months), especially when evaluating for risk of CD relapse. Future studies need to clearly define the study population, recruit larger cohorts, quantify co-existing IBS, and provide a longer follow up period.

The remaining three clinical studies assessing the efficacy of dietary therapies in mild/inactive CD included a LCD (< 84 g per day) [25], a diet low in red meat (≤ 1 serving per month) [21] and an unrefined carbohydrate diet [22]. Study duration was more favourable, ranging from 49 weeks to 2 years, as was the sample size of the studies. Despite this, there was no significant benefit from these diets compared to the control arms in disease activity, relapse rates, biochemical markers of activity, or quality of life indices. The longer study duration in dietary therapy can be offset by diminished compliance to the diet with time and impact efficacy. This highlights one of the challenges of dietary clinical trials in a chronic disease, specifically when assessing its role as a maintenance agent in preventing disease relapse. The duration of the study needs to be adequate to capture relapse of disease, but dietary compliance can drop off beyond 3 months and should be taken into consideration, as demonstrated in prior studies [16, 18, 27, 32].

The impact of dietary therapy was likely attenuated in two studies [21, 25] due to flaws in the study design. The low red meat diet limited the intake of red meat to two meals per week in the control arm. This is a likely change from the habitual diet in some participants, therefore introducing an intervention in the control arm. A true ‘placebo’ arm is not possible in dietary therapy studies, but it is important to ensure that the control arm is close the participants’ habitual diet to prevent confounding impact of any new dietary alteration. The LCD [25] enforced a low dose eight-week steroid course in all participants prior to study entry, which also may have attenuated any rates of relapse amongst both arms of the studies.

### Clinically active CD (induction therapy)

Of the studies reviewed, promising results were found in the MD study that reduced disease activity in group of paediatric patients with mild to moderate disease activity [16]. The outcome lost statistical significance by week 12, possibly due to the small population size in this study. A smaller study of 14 paediatric patients noted high clinical remission rates among all three intervention arms (SCD, modified SCD or wholefoods diet) after a strict SCD for all in the first 2 weeks, which unfortunately is a design flaw and confounds the other two diet arms. In addition, there was no true control arm to the study, therefore limiting the interpretation of the study compared to a habitual diet. Response to a wholefood diet has been shown in a single arm pilot study in children (CD-TREAT), not reviewed here due to the single arm design [36]. Five children undertaking a whole food diet for 8 weeks demonstrated a reduction in disease activity (weighted PCDAI) (*p* = 0.005) and FCP, comparable to that found in children with newly diagnosed CD on EEN [37, 38]. Future well designed studies could provide promising outcomes are required to confirm the impact these dietary interventions.

In the adult population, favourable outcomes were seen in three studies, though the limitations in study design and subsequent validity of outcomes should be noted [18, 28, 32]. The DINE CD study compared two different dietary therapies – the SCD and MD, and although the primary end point was not achieved in assessing superiority of SCD over MD, there was symptomatic response in disease activity (CDAI) in both dietary interventions over 40%. In the absence of a negative control diet, it is not possible to conclude a benefit over the patient’s usual diet. Additionally, the population included was heterogenous and inclusion criteria mandated symptomatic CD based on the CDAI but entry level FCP levels were only minimally elevated in both arms (mean 107 ug/L in SCD and 40 ug/L in the MD arm). Within the subset of patients with elevated CRP and FCP at baseline, both MD and SCD failed to show improvement and not all patients had inflammatory markers reported.

A small pilot study assessing the CDED (29 adult patients), analysed a homogenous population with stricter entry criteria [18]. This did show favourable outcomes but requires validation in a powered randomised controlled trial.

The low IgG4 diet showed significant improvement in clinical activity as measured by the CDAI compared to a control diet [28], though the clinical relevance could be debated given the difference of only 40 points. There was an improvement in quality of life in the intervention group, but no significant difference in inflammatory markers or endoscopic score. There is conflicting evidence in the literature regarding the link between IgG4 levels and dietary modification. It has been postulated that food components in blood stimulate high IgG4 levels and that these in turn may play a role in the inflammatory pathways of IBD, though exact mechanisms are unclear [39]. A large retrospective database of 282 patients found an association of serum IgG4 and disease outcomes in patients with IBD was inconclusive [40]. Testing for IgG4 against foods has now gone out of favour and is no longer recommended as a diagnostic tool [41] due to the disproportionate false positives.

Methodology limitation in these studies included small sample sizes and high dropout rates due to dietary non-compliance or progression of disease. Heterogenous entry criteria is noted in the range in disease activity at baseline, differing usage of concomitant medications, and interventions prior to the study commencement. A true placebo arm in dietary studies is generally not feasible, and some of the studies addressed this by comparing 2 or 3 dietary interventions, though this limits the interpretability of the study. If no difference is noted, it could be due to equal effectiveness of both diets or a type 2 error (i.e. concluding in error that there was no difference when one existed). In some studies, the control arm also had alterations to their diet, therefore introducing a confounding bias as a result of change from the patient’s baseline (habitual) diet. Study duration varied considerably, from 4 weeks to 2 years. Duration of diet studies is contentious, as longer trials are required in a chronic disease such as CD to measure outcomes, but this usually comes at the cost of reduced compliance with the intervention. Future dietary therapy studies need to address some of these limitations to improve reliability of results.

The microbiome has a pivotal role in the pathogenesis and inflammation in CD, and there is growing evidence for the impact of diet on both the composition and function of the microbiome [42–45]. An evolving concept is that of precision nutrition, which is focussed on inter-individual variability in response to diet. It is likely that dietary intervention is more efficacious in some CD patients. Predictors of response include but are not limited to clinical patient factors, their microbiome and metabolomics, individual genetics [44] and various components of food such as food additives. Efficacy of dietary therapy in a more severe phenotype of CD also warrants further exploration, as most studies to date focus on a milder disease phenotype.

The strengths of this study include the meticulous review of the literature in addressing the study question and applying a structured methodology to assessing study bias. Only high-quality studies were included, with no observational studies due to the significant limitations and bias in the latter. The limitations of this review include possible publication bias relating to inclusion of select data by studies, and therefore the increased likelihood of including statistically significant studies. Our systematic review may underestimate the value of dietary therapy due to the innate differences in study design. This includes the lack of true placebo, difficulties in blinding dietary interventions, and lack of reliable tools to measure the variable adherence to the intended intervention.

The review offers a concise and practical summary of clinical trials assessing the efficacy of solid food dietary therapy in the induction of remission and use as maintenance therapy for CD patients. Our findings aim to guide physicians in daily practice when consulting with patients on the role of diet as a therapy for patients with CD.

## Conclusions

There are promising outcomes for the MD and CDED in inducing clinical remission in mild to moderate CD. The results need to be interpreted with caution due to design limitations, such as combining outcomes among CD and UC, and small sample size. Patient satisfaction with dietary therapies has shown adequate tolerability in the short to medium term. Overall, solid food dietary therapy trials are limited by several methodological flaws and future well powered RCTs should be designed to overcome these.

**Table 2:** Outcomes for patients with clinically active CD

Author Country (Year)	Study Design	Participant Age	Study Duration	Study Sample Size	Disease Status at Initiation of Study	Intervention 1 (ITT unless specified)	Control/Intervention 2 (ITT unless specified)	Outcome Measures	Clinical remission (Results)	Other markers of remission and/or disease outcomes (Results)	Inflammatory markers (Results)	Quality of life (Results)	Study authors’ conclusions
***Lewis et al*** ***USA Pennsylvania*** ***(2021)***	Head-to-head randomised trial	> 18 years	12 weeks	ITT analysis (n = 191) PP analysis (limited to participants who reported that they attempted to follow the diet all the time in the week before the week 6 visit) Withdrawals after study commencement: 33 patients prior to week 6 (15 in MD, 18 in SCD), and 37 patients between weeks 6 and 12 (17 in MD, 20 in SCD).	CD diagnosis with mild-moderate symptoms defined by 175 < sCDAI < 400, and ability to complete daily online symptom surveys. Patients with dose changes of CD specific medications or antibiotics in the preceding 2 to 12 weeks* were excluded. *See article for time frames for specific medications	Specific Carbohydrate Diet (SCD) (n = 99)	Mediterranean Diet (MD) (n = 92)	**1. Symptomatic remission** Defined as sCDAI < 150 at week 6, in the absence of initiation or increase of any CD medications. **2. Change in inflammatory markers** Defined by changes in FCP and CRP response. Measured after 6 and 12 weeks on assigned diet.	There was no significant difference between patients in the SCD group, compared to the MD group (SCD 46.5%, MD 43.5%, p = 0.77). There was improvement in the sCDAI, CDAI, short IBDQ, fatigue, sleep interference, pain and social isolation for both study groups (p < 0.02 for all outcomes in both arms).	Not applicable	CRP did not change significantly in either group, from screening to week 6. Reduction in FCP was significant in the SCD group (p = 0.001). Between groups, this was not significant (p = 0.44).	Not applicable	Symptomatic remission was common with both diets, but was not superior with SCD relative to MD. Neither diet was associated with normalisation of CRP.
***Yanai et al*** ***Israel *** ***(2021)***	Open-label randomised trial	18–55 years	24 weeks	ITT analysis (n = 40) PP analysis (N/A) Withdrawals after study commencement: 3 patients in CDED + PEN group due to worsening of disease (2) and lost to follow up (1). 8 patients in CDED group due to worsening of disease (4), intolerance to CDED (2), lost to follow up (1), admission to hospital for colitis (1).	Patients with mild to moderate disease activity, defined by HBI score of 5–14 points. Patients who had worsening disease, or who did not achieve remission or good response (defined as a decrease in HBI of 3 points or more) by week 6, discontinued the study and did not progress to the next phase; treatment was deemed to have failed for these patients for the purposes of the ITT analysis. Patients who did not achieve remission at week 12 or did not achieve a good response did not progress to the maintenance phase of the diet. Use of any other additional therapy was considered treatment failure from that timepoint.	CDED alone (3 phases) (n = 21)	CDED + partial enteral nutrition (PEN) (n = 19)	**1. Clinical remission** Defined as HBI score < 5 at week 6. **2. Corticosteroid-free remission** Proportion of patients in corticosteroid-free remission at weeks 6, 12 and 24 **3. Change in inflammatory markers** Defined by changes in CRP and FCP at weeks 6, 12 and 24. **4. Endoscopic remission** Defined by mucosal healing at weeks 24 to 26 (a SES-CD score of ≤ 3 was considered to indicate endoscopic remission).	68% of patients in the CDED + PEN group and 57% in the CDED group achieved remission by week 6, but there was no significant difference observed between the treatment groups (p = 0.46). Overall, HBI score decreased significantly in all groups between baseline (HBI = 7), week 6 (HBI = 3), 12 (HBI = 2) and 24 (HBI = 2) (p < 0.0001 for all time points), with no difference between groups.	**Corticosteroid-free remission** There was no statistically significant difference between the two groups of patients who had sustained remission. **Endoscopic remission** Among 22 patients with paired colonoscopies at baseline and week 24, median SES-CD score decreased by 5 points (IQR − 6.2 to -1.0); 72.8% decrease from baseline (p = 0.0025). No significant difference was noted in the proportion of patients who achieved endoscopic remission between groups (52% in the CDED + PEN group vs 46% in the CDED alone group; p = 0.70).	A decrease in median CRP from 14.5 to 8.4 was observed at week 24 (p = 0.0098). However, no significant difference was identified between groups. Median FCP concentrations decreased significantly between baseline and week 12, but there was no significant difference between groups.	Not applicable	CDED with or without partial enteral nutrition was effective for induction and maintenance of remission in adults with mild-to-moderate biologic naïve Crohn’s disease and might lead to endoscopic remission. CDED should be assessed in a powered randomised controlled trial.
***El Amrousy et al*** ***Egypt*** ***(2020)***	RCT	12–18 years	12 weeks	ITT analysis (n = 54) PP analysis (same as ITT) Withdrawals after study commencement: 4 patients due to difficulty maintaining diet and failure of attendance, however they were replaced by new patients fulfilling study protocols. Due to difficulty of adhering to diets, 2 patients were unable to complete the trial (treatment arm not specified).	Patients with mild or moderate disease activity, as defined by a PCDAI score of 10–45. Prior to the trial, no changes in IBD medication could be made for at least one month with immunosuppressive medicines and two months with biologics.	Mediterranean diet (MD) (n = 26) KIDMED Score ≥ 8 points throughout entire study period	Standard diet (n = 28) KIDMED score ≤ 7 throughout entire study period	**1. Clinical disease activity** PCDAI questionnaires were completed at each study visit. **2. Biological inflammatory markers** ESR, CRP, serum albumin at each follow-up visit. At weeks 4 and 12, stool calprotectin, serum TNF, IL17, IL10, IL12, IL13 levels were measured. Clinical and laboratory follow up visits/evaluations at weeks 2, 4, 8 and 12.	At week 8, significant clinical remission was achieved in 14 patients undertaking the MD diet compared to only 8 patients undertaking the standard diet (p = 0.04). At the end of the study (week 12), 24 patients in the MD group were in clinical remission with a significantly lower mean PCDAI score compared to 12 patients in the regular diet group (p = 0.02)	Not applicable	Given data for CD and UC patients were combined, most inflammatory markers (CRP, calprotectin, TNF-a, IL17, IL12, IL13) significantly improved with MD.	Not applicable	In children and adolescents adhering to MD for at least 12 weeks, clinical disease activity can be improved. Inflammatory markers are likely to also improve, however studies separating the results of CD and UC patients are required to confirm this.
***Suskind et al*** ***USA Seattle*** ***(2020)***	Head-to-head randomised trial	7–18 years	12 weeks	ITT analysis (n = 14) PP analysis (n = 10) Withdrawals after study commencement: 4 patients due to non-compliance with diet or refusal for ongoing participation.	Mild-moderate CD, defined by PCDAI 15–45. Patients must not have had medication changes for his/her inflammatory bowel disease medications for at least two months prior to enrolment. For the first two weeks, all patients went onto a strict SCD and were then placed onto their randomised diet plan.	**SCD**: Removes all grains, milk products except hard cheeses, sugars outside of honey and most processed foods. (n = 5 for ITT) (n = 4 for PP) **Modified SCD**: SCD with oats and rice (n = 5 for ITT) (n = 4 for PP)	**Whole foods diet**: Eliminating wheat, corn, sugar, milk and food additives (n = 4 for ITT) (n = 2 for PP)	**1. Clinical remission** Defined as PCDAI < 10. **2. Change in inflammatory markers** Clinic follow-up at weeks 2, 4, 8 and 12. Inflammatory labs, and standardised questionnaires, including the PCDAI, were completed during each study visit.	At week 12, all participants who completed the study achieved and maintained clinical remission. **SCD**: ITT analysis showed a decreased PCDAI from 23.5 ± 6.0 at enrolment to 14 ± 17.8 at 2 weeks and 1.9 ± 3.8 at 12 weeks in the SCD group. PP analysis showed a decrease from 21.9 ± 5.5 at enrollment to 6.25 ± 4.8 at 2 weeks and 1.9 ± 3.8 at 12 weeks. **MSCD**: ITT analysis showed a decreased PCDAI from 29.6 ± 9.5 at enrolment to 8.0 ± 5.4 at 2 weeks and 3.1 ± 4.7 at 12 weeks. PP analysis showed a decreased PCDAI from 25.6 ± 8.5 at enrollment to 5.6 ± 1.3 at 2 weeks and 3.1 ± 4.7 at 12 weeks. **Whole food**: ITT analysis showed a decreased PCDAI from 21.6 ± 8.4 at enrolment to 15.0 ± 10.8 at 2 weeks and 1.3 ± 1.8 at 12 weeks. PP analysis showed a decreased PCDAI from 16.25 ± 1.8 at enrollment to 7.5 ± 10.6 at 2 weeks and 1.3 ± 1.8 at 12 weeks. *Note: Overall, small sample sizes prohibited robust determinations of statistical significance*	Not applicable	CRP and ESR improved in each group, with normalisation in both SCD and MSCD groups.	Not applicable	All diets were associated with high and comparable rates of clinical remission. While the MSCD group had normalised and the SCD group had near normalisation of ESR and CRP, the whole foods group did not.
***Gunasekeera et al*** ***London*** ***(2016)***	RCT	Nil age range specified.	4 weeks	ITT analysis (n = 98) PP analysis (n = 76) Withdrawals after study commencement: 17 patients withdrew (unknown reason), 5 patients were lost to follow up.	CD patients with a minimum CDAI score between 80 and 400. A cut-off value of < 150 is regarded as the point where a patient is considered to be in remission. Patients with change in therapy 2 months prior to commencing study were excluded.	**True diet exclusion** Diet excluding the four food types with the highest IgG4 titres (n = 50 for ITT) (n = 39 for PP)	**Sham diet** Diet excluding the four food types with the lowest IgG4 titres (n = 48 for ITT) (n = 37 for PP)	**1. Quality of life** Determined by the SIBDQ **2. Clinical disease activity Determined by CDAI and HBI** **3. Systemic inflammation** Measured by CRP **4. Intestinal inflammation** Measured by FCP At screening visit, blood samples were taken to detect the presence of IgG4 antibodies specific to the food antigens, CRP, stool sample for faecal calprotectin. Each patient also completed a demographic questionnaire, SIBDQ, CDAI and HBI.	There was a significant improvement in CDAI for the true diet exclusion group compared to the sham group, for both ITT (p = 0.009) and PP (p = 0.009) analyses.	Not applicable	No significant improvement. CRP not included in ITT analysis. No significant improvement (p = 0.16). FCP not included in ITT analysis.	There was a significant improvement in SIBDQ for the true diet exclusion group compared to the sham group, for both ITT (p = 0.05) and PP (p = 0.007) analyses.	Dietary modification through identification of offending foods by IgG4 ELISA results in clinical benefit, most likely in those with the most active disease to be a consequence of reducing both systemic and local bowel wall inflammation. IgG4-guided exclusion diet, as an adjunct, can improve quality of life and symptoms in patients with CD.
***Brotherton et al*** ***Virginia *** ***(2014)***	RCT	18–64 years	4 weeks	ITT analysis (n = 7) PP analysis (same as ITT) Withdrawals after study commencement: Nil	Patients with CD, who had 3 ≤ pHBI < 9 and at least 4 weeks of stable pharmacologic therapy. Individuals using biologic drugs were excluded from the study.	High fibre and low refined carbohydrate diet instruction, including consumption of whole wheat bran cereal (n = 4)	Control diet consisting of general dietary instruction (n = 3)	**1. Quality of life ** Baseline and biweekly IBDQ **2. Disease severity assessment** Baseline and weekly telephone interview to score pHBI. **3. Change in inflammatory markers** Baseline and end of study CRP and ESR	The pHBI scores decreased significantly over time in the active wheat bran intervention group, demonstrating improved GI function compared to participants in the attention control group (p = 0.008)	Not applicable	No statistically significant group differences on either CRP (p = 0.125) or ESR (p = 0.788) at 4 weeks	The intervention group had significantly improved IBDQ (p = 0.028) over time than the control group.	Diet modification, specifically an alteration in the types of carbohydrates consumed daily, may be a welcomed complementary therapy for those individuals who suffer lingering GI disruption associated with CD and who desire restoration of continent bowel function.
***Bartel et al*** ***Austria*** ***(2008)***	RCT	Nil age range specified.	24 weeks	ITT analysis (N/A) PP analysis (n = 14) Withdrawals after study commencement: N/A	CD patients with mild-moderate active CD (CDAI 150–220) with ulceration of the left-sided colon or a significant lesion of the small bowel or right-sided colon that was assessable by means of MRI. Patients with used/adjusted dose of steroids over the past 4 weeks, biologics that had been initiated or changed during the previous 3 months, or antibiotics were excluded.	**Initial 6 weeks** Highly restricted diet composed of red meat (pork, beef, lamb), certain sourdough bread, rape oil, and fresh butter, all of which came from intensively monitored organic farming. (n = 5 for PP) Only plain tap water and organic tea were allowed for drinking. **Follow-up period (weeks 6 to 24)** Stepwise add food derived from organic farming, such as local fruits and vegetables, dairy products, beer, wine, honey. Refined sugar and ready-made canned or frozen food were not allowed.	**Initial 6 weeks** Low fat, high carbohydrate diet complete in nutrients. Advised to avoid fibre-rich fruits, vegetables, and red meat. (n = 9 for PP) **Follow-up period (weeks 6 to 24)** Instructed to eat red meat but to avoid fibre-rich and hard fibrous fruits and vegetables.	**1. Endoscopic signs of intestinal inflammation** Assessed at baseline and after 6 and 24 weeks of dietary intervention. **2. Disease activity ** Measured using CDAI **3. Quality of life** Measured using IBDQ **4. Change in inflammatory markers** Measured using CRP, ESR	CDAI improved in both groups to a similar extent. Nil test of significance was undertaken.	**Endoscopic signs of intestinal inflammation** At 6 weeks, endoscopy showed improvement of intestinal lesions in 3 of 4 assessable patients of the active group, and 1 of 9 patients of the control group (p = 0.027). PP analyses only.	Nil change in CRP or ESR. Nil test of significance was undertaken.	IBDQ improved in both groups to a similar extent. Nil test of significance was undertaken.	This study indicates that ingested matter within a Western lifestyle may contribute to the development of CD and that its avoidance may induce intestinal lesions.
***Levenstein et al*** ***Italy *** ***(1985)***	RCT	Nil age range specified.	23–34 months (mean 29 months)	ITT analysis (n = 58) PP analysis (same as ITT) Withdrawals after study commencement: Nil	Patients with non-stenosing, active CD. Exclusion criteria not specified.	Low residue diet (n = 30)	Standard "liberalised" diet (n = 28)	**1. Disease activity** Measured using CDAI **2. Disease outcomes** Based on surgical outcomes of patients.	Nil significant difference in mean CDAI between groups	**Disease outcomes** Nil significant difference in surgical outcomes, other poor outcomes or total outcomes. There was not enough data in the inactive group to draw conclusions in this study.	Not applicable	Not applicable	Nil difference in outcome between the two groups, including symptoms, need for hospitalisation, need for surgery, new complications, or post-operative recurrence. Lifting of dietary restrictions does not cause symptomatic deterioration or precipitate intestinal obstruction in CD.

### Electronic supplementary material

Below is the link to the electronic supplementary material.

Supplementary Material 1

## Data Availability

All data generated or analysed during this study are included in this published article and its supplementary information files.

## Abbreviations

IBD: inflammatory bowel disease
CD: Crohn’s disease
UC: ulcerative colitis
FODMAP: fermentable oligosaccharides, disaccharides, monosaccharides and polyols
PEN: partial enteral nutrition
EEN: exclusive enteral nutrition
MD: Mediterranean diet
SCD: specific carbohydrate diet
CDED: Crohn’s disease exclusion diet
LCD: low carbohydrate diet
IBS: irritable bowel syndrome
IBS: SSS–irritable bowel syndrome severity scoring system
sCDAI: short Crohn’s disease activity index
SIBDQ: short inflammatory bowel disease questionnaire
PCDAI: paediatric Crohn’s disease activity index
SES: CD–simple endoscopic score for Crohn’s disease
HBI: Harvey Bradshaw index
ITT: intention to treat
PP: per protocol
CRP: C–reactive protein
FCP: faecal calprotectin
RCTs: randomised controlled trials
RoB: risk of bias
