# Supplementary material for: The effect of solid food diet therapies on the induction and maintenance of remission in Crohn’s disease: a systematic review
Source: BMC Gastroenterol. 2024 Aug 6;24:250. doi: 10.1186/s12876-024-03315-7 (PMC11302831; doi:10.1186/s12876-024-03315-7)
Supplement: Supplementary file 1 — Supplementary Material 1 [file 12876_2024_3315_MOESM1_ESM.docx]

**APPENDIX 1: SEARCH STRATEGIES**

Search strategy for MEDLINE

1. Crohn disease/ or crohn*.mp.
2. “Crohn’s disease”.kw.
3. (terminal adj2 ileitis).mp.
4. (regional adj2 enteritis).mp.
5. Ileocolitis.mp.
6. Inflammatory bowel diseases/
7. Inflammatory bowel disease/
8. Inflammatory bowel disease*.mp.
9. exp Diet/
10. (diet adj therapy).tw.
11. 1 or 2 or 3 or 4 or 5 or 6 or 7 or 8
12. 9 or 10
13. Remission induction/
14. 11 and 12 and 13

Search strategy for EMBASE

1. Crohn disease/ or crohn*.mp.
2. “Crohn’s disease”.kw.
3. (terminal adj2 ileitis).mp.
4. (regional adj2 enteritis).mp.
5. Ileocolitis.mp.
6. Inflammatory bowel diseases/
7. Inflammatory bowel disease/
8. Inflammatory bowel disease*.mp.
9. exp Diet/
10. (diet adj therapy).tw.
11. 1 or 2 or 3 or 4 or 5 or 6 or 7 or 8
12. 9 or 10
13. Remission/
14. 11 and 12 and 13
15. Limit 14 to (clinical trial or randomised controlled trial or controlled clinical trial or multicentre study)

Search strategy for Cochrane library

1. (inflammatory bowel disease):ti,ab,kw AND (diet*):ti,ab,kw

*In Trials with Gut in Cochrane Groups (Word variations have been searched)*

1. MeSH descriptor: [Inflammatory Bowel Diseases] this term only and with qualifier(s): [diet therapy – DH]

Search strategy for WHO international clinical trials registry platform (ICTRP)

Inflammatory bowel disease OR Crohn's disease in **title**

AND dietary in **intervention**

Recruitment status - ALL

With results only

Search strategy for ANZCTR

Description of intervention(s) / exposure: *Diet*

Intervention code: Treatment: Other + Rehabilitation + *Lifestyle* +Behaviour +other interventions

Keep or delete lifestyle? (Keep diet above)

Allocation to intervention: *Randomised*

Health condition(s) or problem(s) studied: "inflammatory bowel disease" OR "crohn's disease”.

Condition category: *Oral and Gastrointestinal*

Condition code: Crohn’s disease / inflammatory bowel disease (one at a time)

*Note: only words/terms in italics were input into the search engines

Search strategy for ClinicalTrials.gov

Condition: Inflammatory bowel disease

Study type: Interventional

Intervention/treatment - diet therapy OR diets OR diet OR dietary

Outcome measure – induction OR remission

Search strategy for MEDNAR

Inflammatory bowel disease diet

Select – medical (685) > clinical trial (100)

Search strategy for BMC proceedings

"Inflammatory bowel disease" “diet” “remission” “randomised control"
